# The pathway of transmembrane cadmium influx via calcium-permeable channels and its spatial characteristics along rice root

**DOI:** 10.1093/jxb/ery293

**Published:** 2018-08-07

**Authors:** Xiaohui Chen, Younan Ouyang, Yicong Fan, Boyin Qiu, Guoping Zhang, Fanrong Zeng

**Affiliations:** 1 Institute of Crop Science, Zijingang Campus, Zhejiang University, Hangzhou, China; 2 China National Rice Research Institute, Hangzhou, China; 3 Key Laboratory of Crop Breeding in South Zhejiang, Wenzhou Academy of Agricultural Science, Wenzhou Vocational College of Science and Technology, Wenzhou, China

**Keywords:** Calcium-permeable channels, Cd influx, Cd uptake, longitudinal variation, rice

## Abstract

To develop elite crops with low cadmium (Cd), a fundamental understanding of the mechanism of Cd uptake by crop roots is necessary. Here, a new mechanism for Cd^2+^ entry into rice root cells was investigated. The results showed that Cd^2+^ influx in rice roots exhibited spatially and temporally dynamic patterns. There was a clear longitudinal variation in Cd uptake along rice roots, with the root tip showing much higher Cd^2+^ influx and concentration than the root mature zone, which might be due to the much higher expression of the well-known Cd transporter genes *OsIRT1*, *OsNRAMP1*, *OsNRAMP5*, and *OsZIP1* in the root tip. Both the net Cd^2+^ influx and the uptake of Cd in rice roots were highly inhibited by ion channel blockers Gd^3+^ and TEA^+^, supplementation of Ca^2+^ and K^+^, and the plasma membrane H^+^-ATPase inhibitor vanadate, with Gd^3+^ and Ca^2+^ showing the most inhibitory effects. Furthermore, Ca^2+^- or Gd^3+^-induced reduction in Cd^2+^ influx and Cd uptake did not coincide with the expression of Cd transporter genes, but with that of two Ca channel genes, *OsAAN4* and *OsGLR3.4*. These results indicate that Cd transporters are in part responsible for Cd^2+^ entry into rice root, and provide a new perspective that the Ca channels OsAAN4 and OsGLR3.4 might play an important role in rice root Cd uptake.

## Teaser

Investigations reveal that Cd can enter into rice cells via Ca-permeable channels, and the uptake varies longitudinally along the rice root.

## Introduction

As a result of atmospheric deposition, wastewater irrigation, use of metal-containing fertilizers and pesticides, and many other industrial processes, cadmium (Cd) has become one of the most toxic and widespread environmental pollutants in agricultural soil (Rizwan *et al.*, 2016). Being a non-essential element for plants, excess Cd in soils interferes with plant growth and development, reduces crop yield, and accumulates to a high level in plant tissues, thus posing a threat to human and animal health through the food chain (Ahmad *et al.*, 2015). To ensure food safety, breeding ‘low-Cd’ crops has become one of the most important strategies to reduce Cd in crops, for which a fundamental understanding of the Cd uptake mechanism in plant roots would be a critical issue.

Although great progress has been achieved over the last decades, the complex pathways of Cd entry into root cells are still not fully understood. Since Cd is a non-essential element for plants and interferes with the uptake of other ions, it was assumed that Cd could be absorbed into cells by the transporters for essential elements such as Fe^3+^, Zn^2+^, and Mn^2+^, due to the lack of specificity of these transporters (Clemens 2001; Ishimaru *et al.*, 2006; Sasaki *et al.*, 2012). It has been well documented that Cd could enter into root cells through various NRAMP (natural resistance-associated macrophage protein) family members, such as OsNRAMP1, OsNRAMP5, and AtNRAMP6; ZIP (zinc/iron-regulated transporter-like protein) transporters, such as AtIRT1 and TcZNT1/TcZIP4; the low affinity calcium transporters such as TaLCT1; or through YSL (Yellow-Stripe 1-Like) proteins such as ZmYSL1 as Cd chelates (Schaaf *et al.*, 2004; Cailliatte *et al.*, 2009; Lux *et al.*, 2011, and references therein; Sasaki *et al.*, 2012;). Of all the transporters above, OsNRAMP5, which is a major contributor to constitutive Mn^2+^ uptake, was considered to be a major route of Cd uptake from the external environment and entry into cells in rice (Ishimaru *et al.*, 2012; Sasaki *et al.*, 2012). However, a recent study by Takahashi *et al.* (2014) revealed that the knock-down of OsNRAMP5 triggered only ~20% reduction in root Cd content but a significant increase in shoot Cd content in both hydroponic and field trials. These results indicated that there might be other pathways for Cd entry into root cells, apart from the poor selectivity of transition ion transporters. Indeed, some studies suggested that Cd could also possibly be taken up via cation channels, such as K^+^ and Ca^2+^ channels, which are relatively non-selective between cations (White and Broadley, 2003; Lindberg *et al.*, 2004; L.Z. Li *et al.*, 2012). However, the contribution of this pathway to root Cd uptake remains to be further verified. Therefore, more effort is still needed to elucidate thoroughly the mechanism by which Cd is taken up into plant roots and cells.

 With the innovation of non-invasive microelectrode measurements such as the microelectrode ion flux estimation (MIFE) technique (Newman, 2001; Shabala, 2006) and Cd-selective microelectrodes (Piñeros *et al.* 1998), Cd^2+^ fluxes have been characterized in various Cd-hyperaccumulating plants (He *et al.*, 2011, L.Z. Li *et al.*, 2012; Sun *et al.*, 2013). However, little is known about the kinetics of Cd^2+^ ﬂux across root cells in the rice plant, which is considered to be a model among monocots for biological research. Furthermore, as rice is a staple food crop for half of the world’s population, Cd in rice grain has become a major source of dietary Cd intake for this part of the world (Wang *et al.*, 2016). Therefore, the characteristics of the transmembrane Cd^2+^ transport and Cd uptake in different root zones were investigated in this study in the absence and presence of a series of treatments by measuring Cd^2+^ flux, Cd concentration, Cd fluorescence localization, and gene expression. The aim of this study was (i) to reveal the longitudinal variation in Cd^2+^ influx and Cd distribution along rice root and explain how such variation is generated; and (ii) to elucidate whether Cd^2+^ enters into rice root cells through ion channels permeable to Ca^2+^ or K^+^ and explore the candidate transporter genes involved in this process.

## Materials and methods

### Plant materials, growth conditions, and treatments

Rice cultivar IR8, which accumulates high amounts of Cd (Yan *et al.*, 2010), was used in the present study. Seeds were surface sterilized with 12.5% NaClO solution, thoroughly rinsed with tap water, and then soaked in distilled water at 25 °C. Two days later, the seeds were germinated with limited water at 35 °C for another 1 d, and sown on the mesh screen in a container with nutrient solution. All the rice seedlings were grown in a controlled-environmental chamber with a photoperiod of 16 h light/8 h dark, light intensity of 225 ± 25 µmol m^–2^ s^–1^, temperature of 30 °C light/25 °C dark, and relative humidity of 85%. The nutrient solution was made according to Zeng *et al.* (2008). Uniform 10-day-old seedlings were selected for electrophysiological measurements or to be treated with 20 µM CdCl_2_ with/without different blockers for further investigations.

### Microelectrode Cd^2+^ flux measurements

Net Cd^2+^ ﬂuxes were measured from the root epidermis of rice seedlings using non-invasive ion-selective vibrating microelectrodes (the MIFE technique, University of Tasmania, Hobart, Australia). The Cd ion-selective microelectrodes with tip diameter of 2–3 µm were manufactured and silanized with tributylchlorosilane (Cat. No. 90796; Sigma-Aldrich, Steinem, Switzerland), and then back-filled with an electrolyte buffer (0.1 mM KCl plus 10 mM CdCl_2_) and front-filled with an ion-selective Cd^2+^ cocktail made up with Cd^2+^ ionophore I, potassium tetrakis, and 2-nitrophenyl octyl ether (Cat. Nos 20909, 60588, and 73732; Sigma-Aldrich) according to Piñeros *et al.* (1998). The well-filled microelectrodes were equilibrated in basic salt medium solution (BSM; 0.5 mM KCl plus 0.10 mM CaCl_2_, buffered with 5 mM MES and 2 mM Tris at pH 5.6) for 1 h and calibrated in 5, 10, and 20 µM Cd in the absence and presence of either pharmaceutical prior to the Cd^2+^ ﬂux measurement. Only electrodes with Nernstian slopes ≥25 mV and correlation >0.9990 were used. Details on measuring ion flux have been described previously (Newman, 2001; Shabala, 2006).

### Experimental protocols for MIFE measurements

#### Measurement of Cd^2+^ flux along the rice root

Cd^2+^ flux profiles along rice root were measured after 1 h incubation in BSM containing 20 µM CdCl_2_, Root scanning started from the root cap and was carried out with 0.1 mm increments between 0 mm and 1 mm, 0.5 mm increments between 1 mm and 10 mm, and 1.0 mm increments between 10 mm and 20 mm, with net ion fluxes measured for 1 min at each point. Five individual seedlings were measured for each treatment.

#### Transient ion flux kinetics

Roots (≥5 cm) of intact seedlings were mounted in a horizontal chamber filled with 30 ml of BSM 1 h prior to measurements. Net ion fluxes were measured for 5 min under the control condition (BSM) to record the steady control flux values. Subsequently, 2 ml or 10 ml of CdCl_2_ stock (80 µM, made up in the background of BSM) was gently added to the chamber to yield the final Cd^2+^concentration of 5 µM or 20 µM, and the transient ion flux responses were measured for another 30 min. The period of time for mixing the solution (~2 min) was omitted from the data analysis and figures. Net ion ﬂuxes were measured in either the elongation zone (EZ, ~2 mm from the root cap, without root hair) or the mature zone (MZ, ~10 mm from the root cap, with root hair). Six individual seedlings were measured for each treatment.

#### Pharmacological measurements

In pharmacological experiments, plants roots were pre-treated for 1 h prior to measurements with 30 ml of one of the following solutions: 100 µM sodium orthovanadate (vanadate); 20 mM tetraethylammonium chloride hydrate (TEA^+^); 100 µM GdCl_3_ (Gd^3+^); 5 mM CaCl_2_; or 10 mM KCl. Net Cd^2+^ fluxes were first measured for 5 min under the condition with either pharmacological treatment to record the steady control flux values. Subsequently, 10 ml of CdCl_2_ stock (80 µM, made up in the background of BSM with each corresponding pharmaceutical) was gently added to the chamber to yield the final Cd concentration of 20 µM, and the transient ion flux responses were measured for another 30 min. All the above pharmacological solutions were made up in the background of BSM, and buffered with MES–Tris (5 mM MES, 2 mM Tris base) at pH 5.6. Five individual seedlings were measured for each treatment.

### Fluorescence labeling of Cd in root

Rice roots were pre-treated with different pharmaceuticals for 1 h, and then given the appropriate volume of Cd stock to yield the final Cd concentration of 20 µM. Rice roots were subsequently treated for 24 h prior to measurements. Cadmium stock was made up in the background of BSM with each corresponding pharmaceutical, and the one made up in the background of BSM only was used as the control. The localization of Cd in rice roots was investigated using the Cd Probe Leadmium Green AM dye (Molecular Probes, Invitrogen, Calsbad, CA, USA) according to L.Z. Li *et al.* (2012) with some modifications. Briefly, a stock solution of fluorescent dye was made by adding 50 ml of DMSO to one vial of Leadmium Green AM. The stock dye solution was then diluted 1:10 with 0.85% NaCl prior to being used. Root segments of 5 mm long from the root cap were thoroughly immersed in diluted dye solution for 1.5 h in the dark. The root segments were rinsed sequentially with 0.85% NaCl, 1 mM Na_2_EDTA, and distilled water, and subsequently slowly shaken in 0.85% NaCl solution for 24 h to get rid of all Cd ions from the root surface. Thereafter, the thoroughly washed root segments were observed under a ﬂorescence microscope (ECLIPSE, Nikon, Japan) with excitation at 488 nm and emission at 500–550 nm. Images were taken with a megapixel digital color camera (Leica DFC425C, Leica Microsystems) and images were acquired using ACDsee software (ACDSee Pro 8, ACD Systems International Inc., Canada). All the features of the camera were set to constant values for each image as follows: exposure time 1.3 s for fluorescence, gain 1.5×, saturation 1.5×, and gamma 1.0×. Each test was repeated at least eight times. The fluorescence intensity was calculated with Image J software (version 1.8.0, National Institutes of Health, USA).

### Determination of root Cd concentration by ICP-MS

The concentration of Cd in rice roots pre-treated with different pharmaceuticals plus 20 µM Cd (see the details in the section on Cd fluorescence labeling) for 3 d was investigated by the inductively coupled plasma mass spectrometry (ICP-MS) technique. Prior to the determination, the roots from each treatment were immersed with 1 mM Na_2_EDTA for 15 min to remove the metal ions from the root surface, and washed thoroughly with double-distilled water. Thereafter, root segments 0–5, 5–10, 10–15, and 15–20 mm from the root cap and the bulk roots were separately collected and oven dried at 70 °C. The weighted dry samples were wet-digested with HNO_3_ plus HClO_4_ (HNO_3_:HClO_4_ =4:1). The resulting clear solutions were diluted with Mili Q water with a ratio of 1:4. Cd concentration was determined using the NexION300X (PerkinElmer, USA) with radial conﬁguration.

### RNA extraction and qRT-PCR

The transcript levels of genes involved in Cd transmembrane transport, such as *OsIRT1*, *OsNRAMP1*, *OsNRAMP5*, and *OsZIP1*, were determined in both the root tip (0–5 mm from the root cap) and the MZ (15–20 mm from the root cap) of rice root. At 0 h, 3 h, and 3 d of 20 µM Cd treatment, root segments from the root tip and the MZ of IR8 were collected and immediately frozen in liquid nitrogen for total RNA extraction. Three biological replicates were measured for each treatment.

To investigate the impact of pharmaceuticals on the expression of genes involved in Cd, Ca, and K transmembrane transport, the seedlings of IR8 were treated with 20 µM Cd and with or without different pharmaceuticals as described in the Cd fluorescence labeling experiment. At 3 h and 3 d of Cd treatment, the bulk of roots from two seedlings were collected and immediately frozen in liquid nitrogen for total RNA extraction. Three biological replicates were measured for each treatment.

Total RNA was isolated using the MiniBEST Plant RNA Extraction Kit (TaKaRa, Japan), and quantitative real-time PCR (qRT-PCR) was performed using Light Cycler 480 II (Roche, Swiss Confederation) with the iTaq™ Universal SYBR Green Spermix (Bio-Rad Laboratories, USA). Primer sequences for qRT-PCR are listed in Supplementary Table S1 at *JXB* online. Three technical replicates were performed for each biological replicate. The relative gene expression was calculated based on the 2^-△△Ct^ method using *OsActin* as the internal standard (Livak and Schmittgen, 2001).

### Data analysis

Statistical analysis was performed by a statistical package IBM SPSS Statistics 20 (IBM, New York, USA). All data in the ﬁgures and table are given as means ±SE. The signiﬁcant difference between means was evaluated by ANOVA test. Significant differences among the means were compared using Tukey’s multiple range tests.

## Results

### Profiles of net Cd^2+^ flux along rice roots

**Fig. 1.**
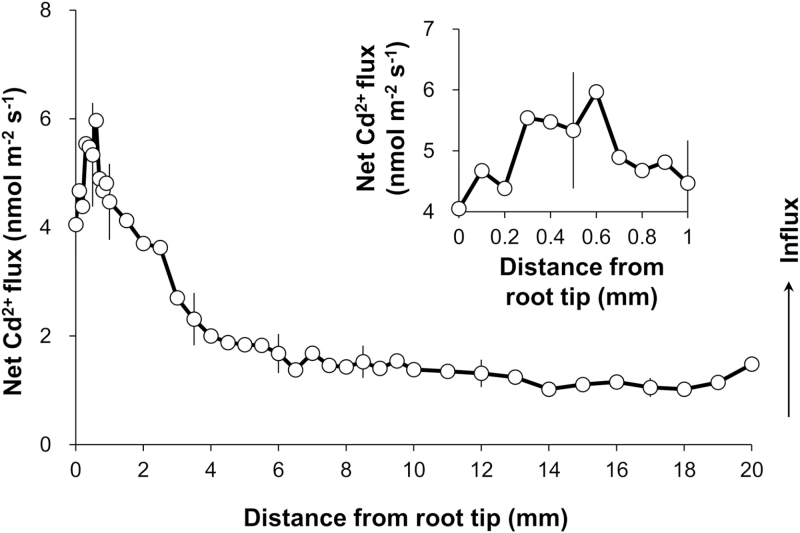
Net Cd^2+^ ﬂux proﬁles along the root axis of cultivar IR8. Net Cd^2+^ ﬂuxes were measured after 1 h exposure to 20 µM CdCl_2_ with 0.1 mm increments between 0 mm and 1 mm, 0.5 mm increments between 1 mm and 10 mm, and 1.0 mm increments between 10 mm and 20 mm, starting from the root cap. The insert displays the close-up view of net Cd^2+^ ﬂux along the root segment between 0 mm and 1 mm. At each position, an average Cd^2+^ ﬂux was measured for 1 min before the electrode was repositioned. Data are means ±SE (*n*=5). For all MIFE data, the sign convention is ‘influx positive’.

To verify whether different root zones would show different Cd^2+^ uptake ability, transient Cd^2+^ flux was measured from different regions along the root axis (0–20 mm) after 1 h exposure to 20 µM CdCl_2_ (Fig. 1). Net Cd^2+^ influx was observed at all positions examined along the rice root, which was saturated at 0.6 mm and started to stabilize ~6 mm from the root cap. The root tip (including the meristem and EZs) showed much stronger Cd^2+^ influx than the MZ, with the largest Cd^2+^ influx of ~6 nmol m^–2^ s^–1^ measured at 0.6 mm from root cap. This is ~4-fold greater than average Cd^2+^ influx in the MZ (10–20 mm).

### Net Cd^2+^ fluxes of rice root epidermal cells under different Cd concentrations

**Fig. 2.**
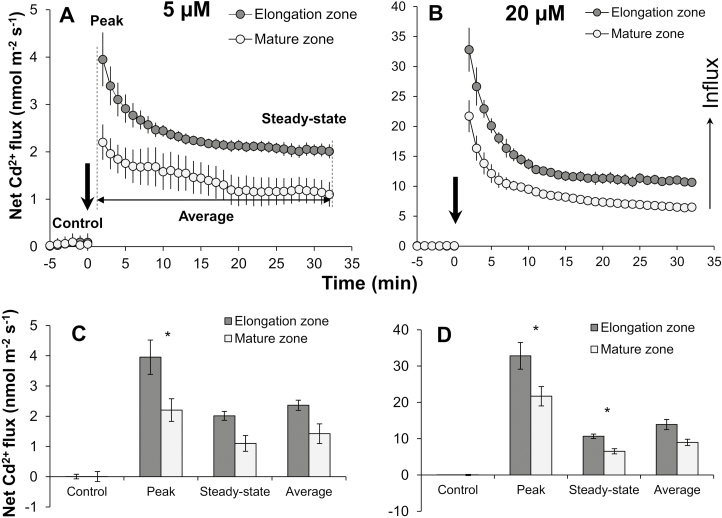
Kinetics of net Cd^2+^ ﬂuxes measured from the elongation zone (EZ; ~2 mm from the root cap, without root hair) and mature zone (MZ; ~10 mm from the root cap, with root hair). (A and B) Transient Cd^2+^ fluxes in response to 5 µM or 20 µM CdCl_2_; Cd treatments were applied at time 0 as indicated by arrows. (C and D) Initial Cd^2+^ flux (under control conditions before CdCl_2_ treatments), magnitude of Cd^2+^ influx (immediately after CdCl_2_ treatments), steady-state Cd^2+^ influx (at the end of the measurements), and average Cd^2+^ influx (measured over the 30 min after CdCl_2_ application) as shown in (A). Data are means ±SE (*n*=6). An asterisk shows the significant difference between two root zones at *P*<0.05.

Prior to adding CdCl_2_ treatments, Cd^2+^ fluxes were kept at ~0 nmol m^–2^ s^–1^ (Fig. 2), indicating that no Cd uptake occurred under the control condition. However, external CdCl_2_ treatments resulted in immediate Cd^2+^ influx from the rice root surface (Fig. 2A, B). Also, a clear dose–response relationship was found between the external Cd concentration and magnitude of Cd^2+^ influx, with a >8-fold larger magnitude of Cd^2+^ flux observed under 20 µM CdCl_2_ (32.8 nmol m^–2^ s^–1^ at the EZ and 21.7 nmol m^–2^ s^–1^ at the MZ) than under 5 µM CdCl_2_ (3.9 nmol m^–2^ s^–1^ at the EZ and 2.2 nmol m^–2^ s^–1^ at the MZ) (Fig. 2C, D). A similar difference was also found for the mean Cd^2+^ influx over the total measurement time of 30 min. In addition, the sharp increase in net Cd^2+^ influx after Cd addition was very short lived and declined to the steady-state value in 20–25 min.

As expected, a significant difference in the net Cd^2+^ influx was found between the EZ and MZ, regardless of the external Cd concentration. The EZ showed a >1.5-fold higher magnitude, mean, or steady-state value of Cd^2+^ influx than the MZ, in agreement with the results of the Cd^2+^ flux profile along the root (Fig. 1).

### Net Cd^2+^ fluxes from rice root in the presence of a proton pump inhibitor and ion channel blockers

**Fig. 3.**
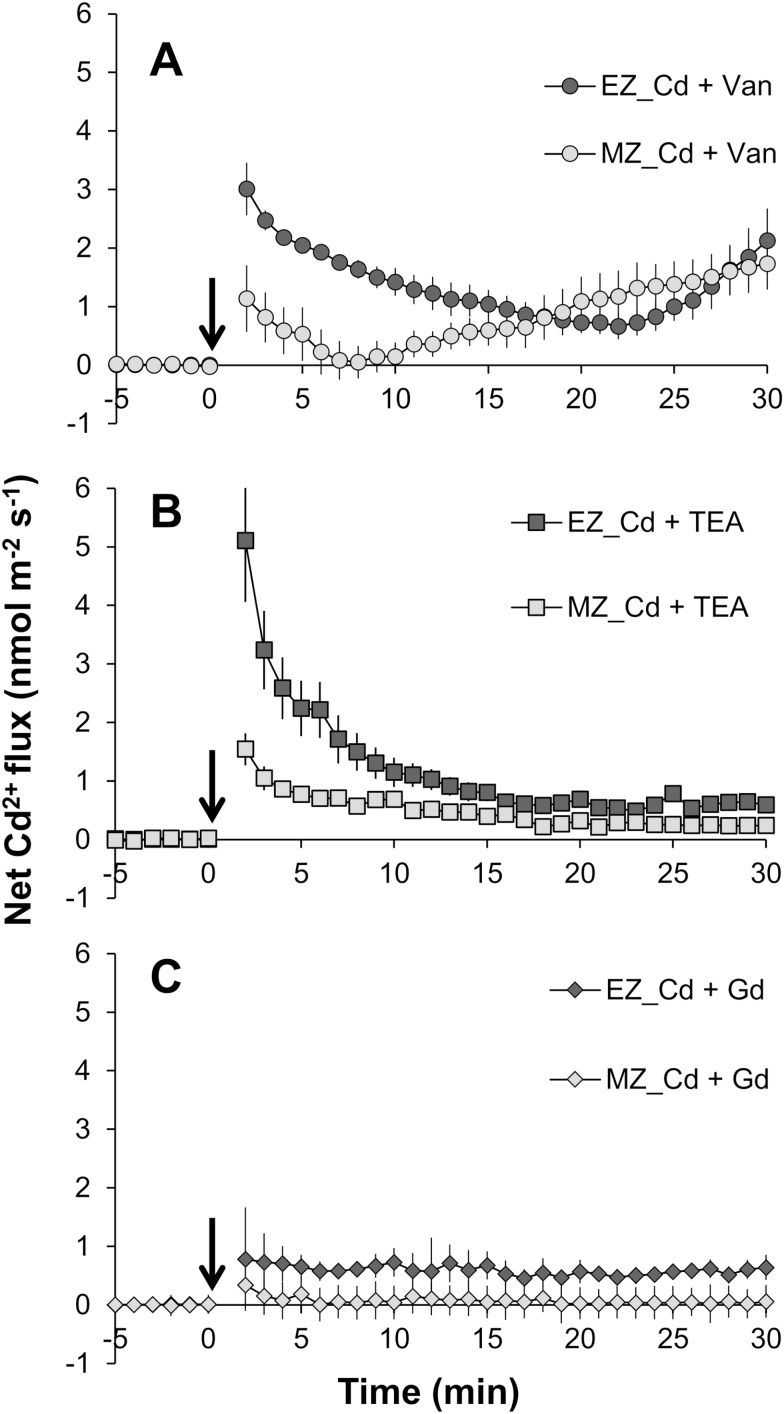
Transient Cd^2+^ ﬂuxes in response to 20 µM CdCl_2_ treatment (added at time zero) from the elongation EZ; ~2 mm from the root cap, without root hair) and mature zone (MZ; ~10 mm from the root cap, with root hair) of rice roots pre-treated with 100 µM vanadate (A), 20 mM TEA^+^ (B), or 100 µM Gd^3+^ (C). Roots were pre-treated with various inhibitors for 1 h before the CdCl_2_ solution (prepared in the background of each inhibitor) was added. Data are means ±SE (*n*=5).

The effects of a proton pump inhibitor and channel blockers on Cd^2+^ flux kinetics were studied to reveal the possible pathways mediating root Cd uptake. None of the inhibitors used in the present study significantly affected the control ion flux after 1 h of incubation (Fig. 3; Table 1).

**Table 1.** Mean control, peak, steady-state, and average Cd^2+^ flux of rice roots pre-treated with different pharmacological agents

	Root zone	Cd fluxes under different pharmacological treatments (nmol m^–2^ s^–1^)				
		Cd+vanadate	Cd+TEA	Cd+Gd	Cd+K	Cd+Ca
Control	EZ	0.005 ± 0.009	0.006 ± 0.002	0.006 ± 0.038	0.008 ± 0.006	0.005 ± 0.000
		(–0.5)	(–0.4)	(–0.4)	(0.0)	(–0.8)
	MZ	0.003 ± 0.098	0.004 ± 0.063	0.003 ± 0.136	0.003 ± 0.086	0.003 ± 0.149
		(–0.2)	(–0.1)	(–0.5)	(–0.2)	(–0.6)
Peak	EZ	3.009 ± 0.449	5.106 ± 3.156	0.781 ± 0.883	5.717 ± 1.005	1.151 ± 0.443
		(–3.4**)	(–2.7*)	(–5.4***)	(–2.5*)	(–4.8***)
	MZ	1.138 ± 0.856	1.541 ± 0.275	0.341 ± 0.438	3.438 ± 1.493	0.019 ± 0.283
		(–4.2**)	(–3.8**)	(–6.0***)	(–2.6*)	(–10.2***)
Steady-state	EZ	2.129 ± 0.544	0.591 ± 0.137	0.639 ± 0.216	1.389 ± 0.427	0.230 ± 0.013
		(–2.4*)	(–4.2**)	(–4.1**)	(–3.0**)	(–5.6***)
	MZ	1.734 ± 0.662	0.239 ± 0.108	0.059 ± 0.279	1.212 ± 0.090	0.727 ± 0.263
		(–1.8*)	(–4.7***)	(–6.7***)	(–2.3*)	(–3.1**)
Average	EZ	1.375 ± 0.250	1.199 ± 0.410	0.596 ± 0.240	1.842 ± 0.536	0.395 ± 0.097
		(–3.4**)	(–3.6**)	(–4.6***)	(–2.9*)	(–5.2***)
	MZ	0.832 ± 0.548	0.482 ± 0.100	0.073 ± 0.292	1.287 ± 0.337	0.511 ± 0.180
		(–3.4**)	(–4.2**)	(–6.9***)	(–2.8*)	(–4.1**)

Control, Cd^2+^ flux under control conditions before CdCl_2_ treatments; Peak, Cd^2+^ influx immediately after CdCl_2_ treatments; Steady-state, Cd^2+^ influx at the end of the measurements; Average, Cd^2+^ influx measured over the 30 min after CdCl_2_ application. Cd+vanadate, 20 µM CdCl_2_ with pre-treatment with 100 µM vanadate; Cd+TEA, 20 µM CdCl_2_ with pre-treatment with 20 mM TEA^+^; Cd+Gd, 20 µM CdCl_2_ with pre-treatment with 100 µM Gd^3+^; Cd+K, 20 µM CdCl_2_ with pre-treatment with 10 mM K^+^; Cd+Ca, 20 µM CdCl_2_ with pre-treatment with 5 mM Ca^2+^. Cd^2+^ flux data are the mean ±SE (*n*=5). Data in parentheses are the fold changes of each pharmacological treatment relative to Cd treatment only, fold change=log_2_^[(Cd2+ flux)^_pharmacological treatment_/^(Cd2+ flux)^_Cd only_^]^. *, **, or *** represents the significance between the treatment with CdCl_2_ plus various pharmaceuticals and the treatment with CdCl_2_ only at *P* <0.05, 0.01, or 0.001.

Root pre-treatment in 100 µM vanadate, a well-known inhibitor of plasma membrane (PM) H^+^-ATPase, caused reduction of the net Cd^2+^ influx response to 20 µM CdCl_2_ treatment, leading to a reduction in the magnitude of Cd^2+^ influx by 3.4-fold for the EZ and 4.2-fold for the MZ, the steady-state Cd^2+^ influx by 2.4-fold for the EZ and 1.8-fold for the MZ, and the mean Cd^2+^ influx by 3.4-fold for both the EZ and MZ (Fig. 3A; Table 1). However, such an inhibitory effect of vanadate was short lived and started to recover ~20 min after adding Cd treatment for the EZ and only 5 min for the MZ.

TEA^+^ is a known blocker of K^+^-selective channels (Maathuis and Sanders, 1996). Pre-treatment with 20 mM TEA^+^ caused a significant decrease of the Cd^2+^ influx, with the magnitude of Cd^2+^ influx reduced by 2.7-fold for the EZ and 3.8-fold for the MZ, the steady-state Cd^2+^ influx reduced by 4.2-fold for the EZ and 4.7-fold for the MZ, and the mean Cd^2+^ influx reduced by 3.6-fold for the EZ and 4.2-fold for the MZ (Fig. 3B; Table 1).

Gd^3+^ is a known blocker of non-selective cation channels (NSCCs), which are known to be permeable to cations such as Ca^2+^, K^+^, and Na^+^ (Demidchik *et al.*, 2002). In comparison with vanadate and TEA^+^, pre-treatment with 100 µM Gd^3+^ induced the largest inhibitory effect on the response of Cd^2+^ flux (Fig. 3C; Table 1). It totally blocked the Cd^2+^ influx to <1 nmol m^–2^ s^–1^ in both the EZ and MZ. The magnitude, steady-state, and mean Cd^2+^ influx were reduced by 4.1- to 5.4-fold for the EZ and by 6.0- to 6.9-fold for the MZ.

Moreover, like the performance under the treatment with Cd only, the EZ also showed much higher net Cd^2+^ influxes than the EZ under treatments with all three inhibitors, with a 1.2- to 10.8-fold difference in the magnitude, steady-state, or mean value of the Cd^2+^ influx.

### Net Cd^2+^ flux changes under elevated external K and Ca concentration

**Fig. 4.**
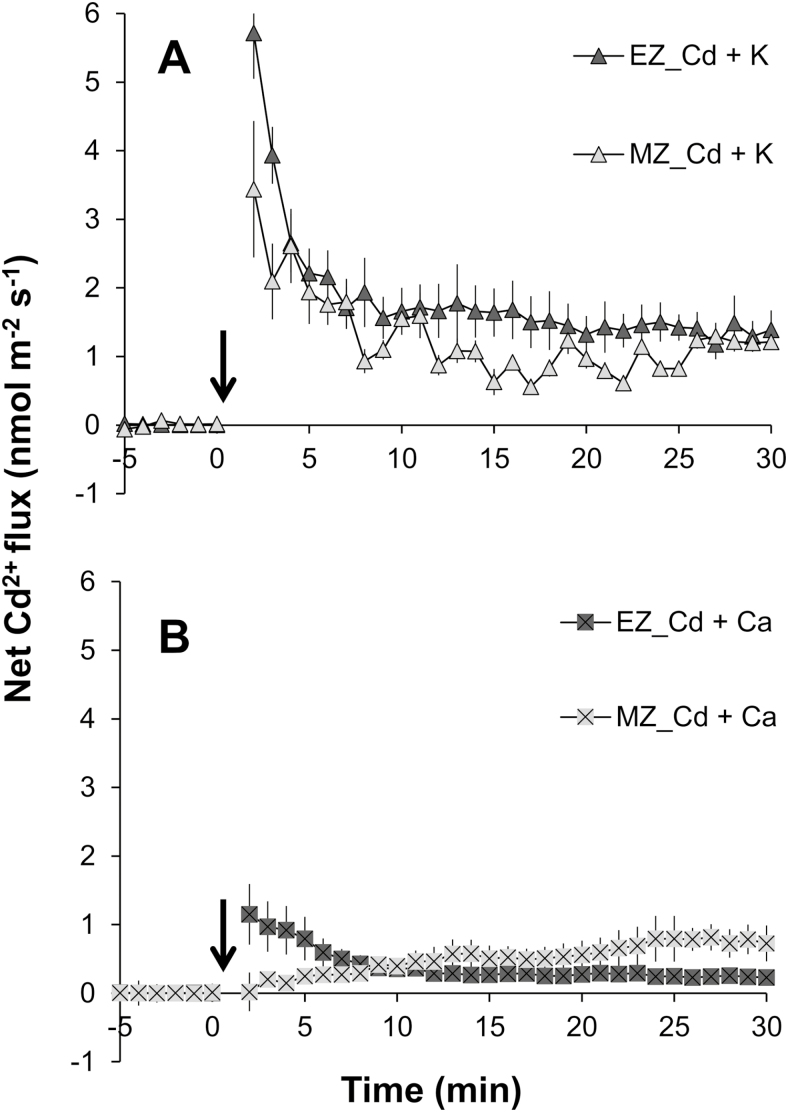
Transient Cd^2+^ ﬂuxes in response to 20 µM CdCl_2_ treatment (added at time zero) from the elongation zone (EZ; ~2 mm from the root cap, without root hair) and mature zone (MZ; ~10 mm from the root cap, with root hair) of rice roots pre-treated with 10 mM K^+^ (A) or 5 mM Ca^2+^ (B). Roots were pre-treated with solution containing high K^+^ or Ca^2+^ concentration for 1 h before the CdCl_2_ solution (prepared in the background of high K^+^ or Ca^2+^ concentration). Data are means ±SE (*n*=5).

It has been well documented that Cd significantly interferes with the uptake and accumulation of various nutrients in plant tissues, including K^+^ and Ca^2+^ (Chang *et al.*, 2003; S. Li *et al.*, 2012; Farooq *et al.*, 2016). Conversely, the supplementation of K^+^ and Ca^2+^ was also reported to reduce Cd accumulation efficiently in plant tissues (Ahmad *et al.*, 2015, 2016). To examine whether the supplementation of K^+^ and Ca^2+^ interfered with the kinetics of net Cd^2+^ influx, rice roots were pre-treated with 10 mM K^+^ or 5 mM Ca^2+^ for 1 h before measuring the net Cd^2+^ flux. Pre-treatment with 10 mM K^+^ strongly reduced the net Cd^2+^ influx into the rice root, causing an ~2.5-fold reduction in the magnitude of Cd^2+^ influx and an ~3.0-fold reduction in the mean Cd^2+^ influx for both the EZ and MZ in comparison with the basic K^+^ level of 0.5 mM (Fig. 4A; Table 1). Surprisingly, the effect of 5 mM Ca^2+^ on Cd^2+^ influx was different between the EZ and MZ (Fig. 4B; Table 1). The pre-treatment with 5 mM Ca^2+^ reduced the Cd^2+^ influx in the EZ by ~5.0-fold. However, the Cd^2+^ flux response to 20 µM CdCl_2_ in the MZ was altered by the pre-treatment with 5 mM Ca^2+^. At the beginning of Cd application, the net Cd^2+^ influx was completely inhibited, whereas it was slowly recovered with extension of the measuring time.

### Total Cd concentration

**Fig. 5.**
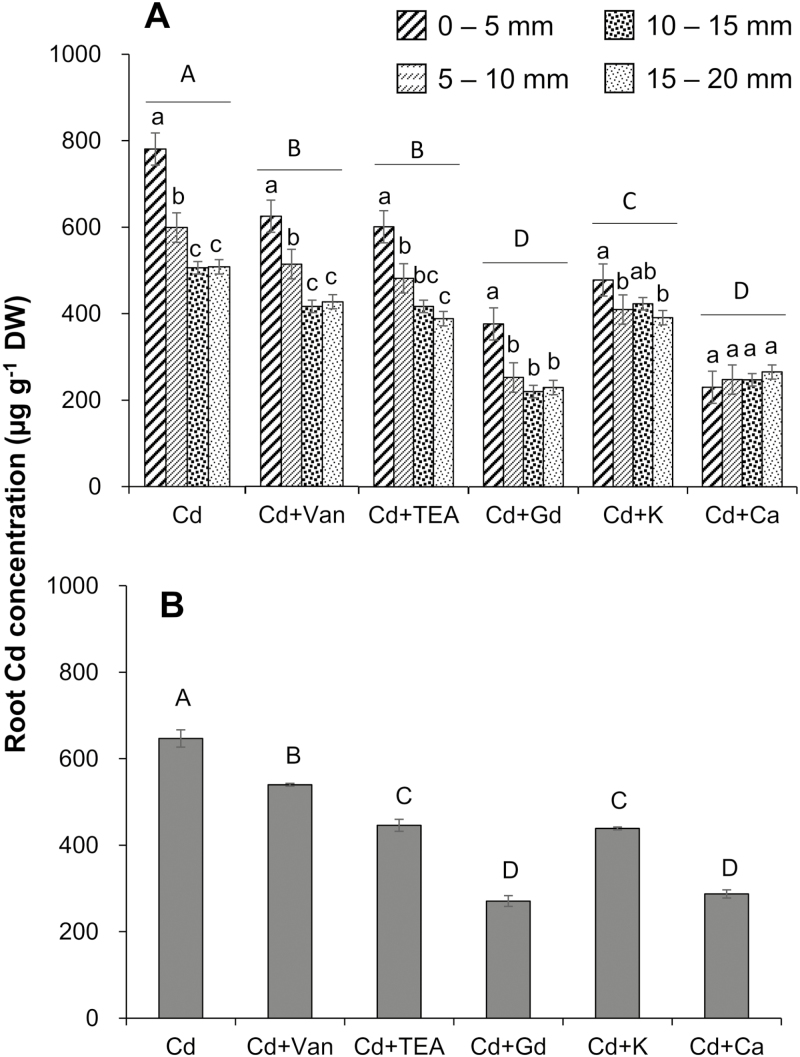
Total Cd concentration in rice root segments (0–5, 5–10, 10–15, and 15–20 mm from the root cap (A) and bulk roots (B) after 3 d of 20 µM CdCl_2_ treatment without or with 100 µM vanadate, 20 mM TEA^+^, 100 µM Gd^3+^, 10 mM K^+^, or 5 mM Ca^2+^. Roots were pre-treated with various pharmaceuticals for 1 h before the CdCl_2_ solution (prepared in the background of each pharmacological reagent) was added. Data are means ±SE (*n*=5). Different lower case letters represent a significant difference between root segments at *P*<0.05; different upper case letters represent the significant difference between treatments at *P*<0.05.

Total Cd concentration was determined in either root segments or the bulk roots of rice seedlings pre-treated with different pharmacological agents after 3 d of Cd exposure to verify the results of MIFE measurements (Fig. 5). Results from Cd determination were mostly in agreement with MIFE data. The root tip (0–5 mm from root cap) had a significantly higher Cd concentration than those segments far away from it (5–10, 10–15, and 15–20 mm from the root cap) (*P*<0.05; Fig. 5A). Pre-treatment with vanadate, TEA^+^, Gd^3+^, K^+^, and Ca^2+^ significantly restricted the accumulation of Cd in rice roots, both in each root segment and in the bulk roots (Fig. 5). Their inhibitory effects on Cd accumulation were in the order of Gd^3+^ (58.1%) ~Ca^2+^ (55.6%) >K^+^ (32.2%) ~TEA^+^ (31.1%) >vanadate (16.5%) (Fig. 5B).

### The distribution of Cd along the rice root

**Fig. 6.**
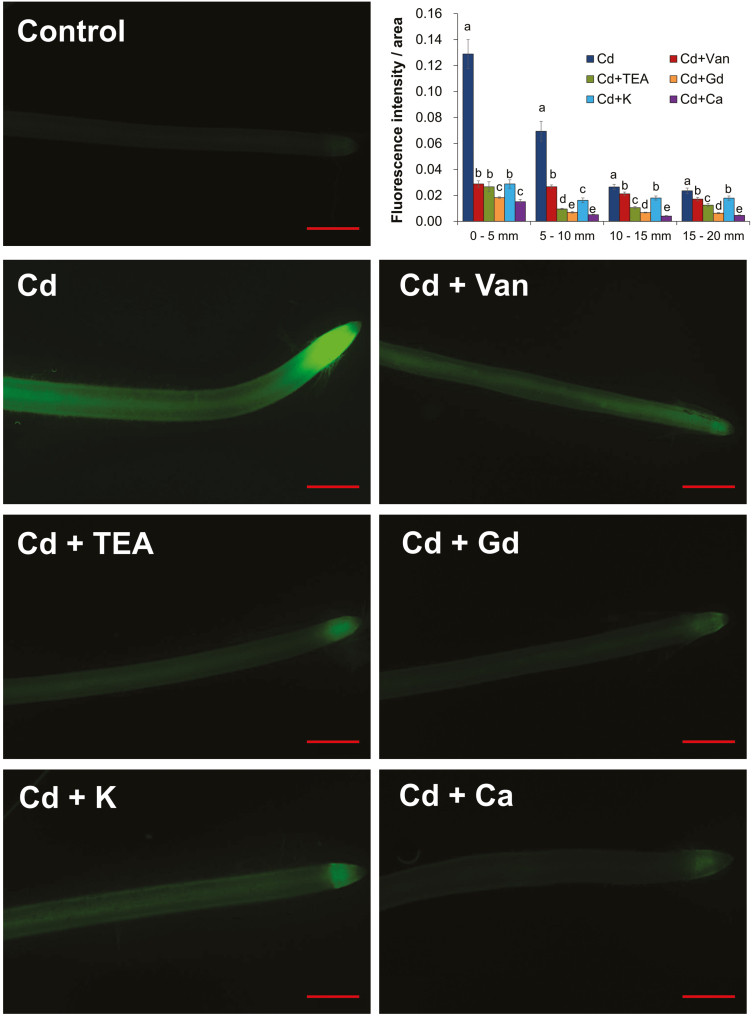
Cd^2+^ accumulation in rice root, visualized by fluorescent imaging using Leadamium Green AM dye. Control, root without CdCl_2_ treatment; Cd, roots treated with 20 µM CdCl_2_ only for 24 h; Cd + Van, Cd + TEA, Cd + Gd, Cd + K or Cd + Ca: roots pre-treated with 100 µM vanadate, 20 mM TEA^+^, 100 µM Gd^3+^, 10 mM K^+^, or 5 mM Ca^2+^, respectively for 1 h before the CdCl_2_ solution (prepared in the background of each pharmaceutical) was added. Green ﬂuorescence in the image represents the binding of the dye to Cd. One (of 8–12) representative images is shown for each treatment. Scale bar=500 µm. Data are means ±SE (*n*=8–12). Different lower case letters represent a significant difference between pharmacological treatments at *P*<0.05.

The effect of inhibitors and nutrients on Cd^2+^ distribution along rice roots (~5 mm long from the root cap) was further investigated using Leadmium Green AM dye after 24 h of Cd exposure (Fig. 6). One hour after the incubation with the fluorescent dye, a clear and bright green fluorescence was observed in the roots treated with 20 µM Cd only (Fig. 6). Pre-treatment with ion channel blockers TEA^+^ and Gd^3+^ suppressed Cd green fluorescence in rice roots (Fig. 6), with the NSCC blocker Gd^3+^ showing a much stronger inhibitory effect than the potassium-selective channel blocker TEA^+^. Supplementation of K^+^ and Ca^2+^ also caused significant reduction in the Cd green fluorescence in rice roots (Fig. 6), but much greater inhibition was seen for Ca^2+^ than for K^+^. Pre-treatment with vanadate showed much less impact on Cd green fluorescence in rice roots compared with the other pharmacological treatments (Fig. 6), in agreement with the results of the Cd concentration (Fig. 5). These results suggest that Cd exhibits high affinity for Ca^2+^-binding sites over K^+^-binding sites during transport across the PM. In addition, a stronger intensity of Cd green fluorescence was observed close to the root cap, regardless of pre-treatments, in accordance with the results of the Cd^2+^ flux profile along the root in our study Fig. 1 or in previous studies (Piñeros *et al.*, 1998; Sun *et al.*, 2013).

### Gene expression at different root zones

**Fig. 7.**
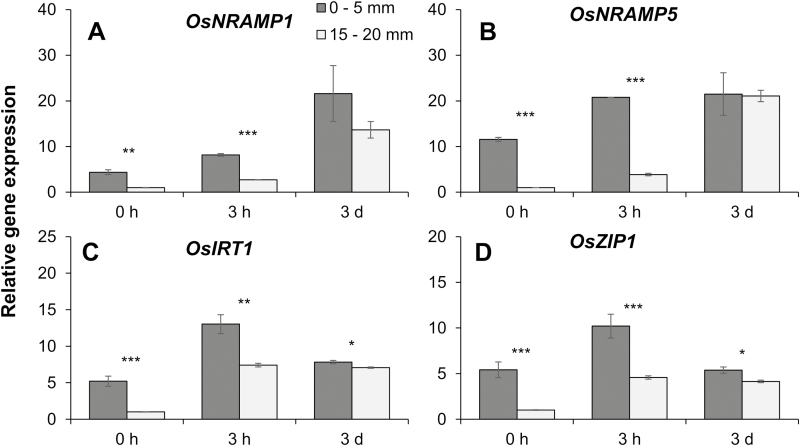
Gene expression of Cd transporters in rice root tip (0–5 mm from the root cap) and mature (15–20 mm from the root cap) zones prior to (0 h) or after onset of 20 µM CdCl_2_ for 3 h and 3 d. Gene expression in the mature zone at 0 h was normalized to 1, and gene expression in the other root tissues or at the other time were all compared relative to it. Data are means ±SE (*n*=3 biological replicates). *, **, or *** represent a significant difference between two root zones at *P*<0.05, 0.01, or 0.001.

The measurements of MIFE (Figs 1, 2), ICP-MS (Fig. 5), and Leadmium Green AM dying (Fig. 6) indicated that there was a significant difference in Cd influx and accumulation between the root tip and the MZ. To reveal the reason for such a difference, the expression level of genes for Cd transport was investigated in the root tip (0–5 mm from the root cap) and the MZ (15–20 mm from the root cap). As expected, the expression of *OsIRT1*, *OsNRAMP1*, *OsNRAMP5*, and *OsZIP1* was induced by 20 µM Cd (Fig. 7), regardless of root zones. Surprisingly, the transcript levels of all these genes were much higher in the root tip than in the MZ prior to or at 3 h after Cd treatment (Fig. 7). With the exposure time of Cd treatment increasing to 3 d, although the difference in gene expression between the root tip and the MZ was greatly reduced, the transcript levels of *OsIRT1* and *OsZIP1* were still significantly higher in the root tip than in the MZ. These results indicated that the root tip had much greater capacity for Cd transport than the MZ, which consequently led to more Cd influx and accumulation in the root tip.

### Gene expression under pharmacological treatments

**Fig. 8.**
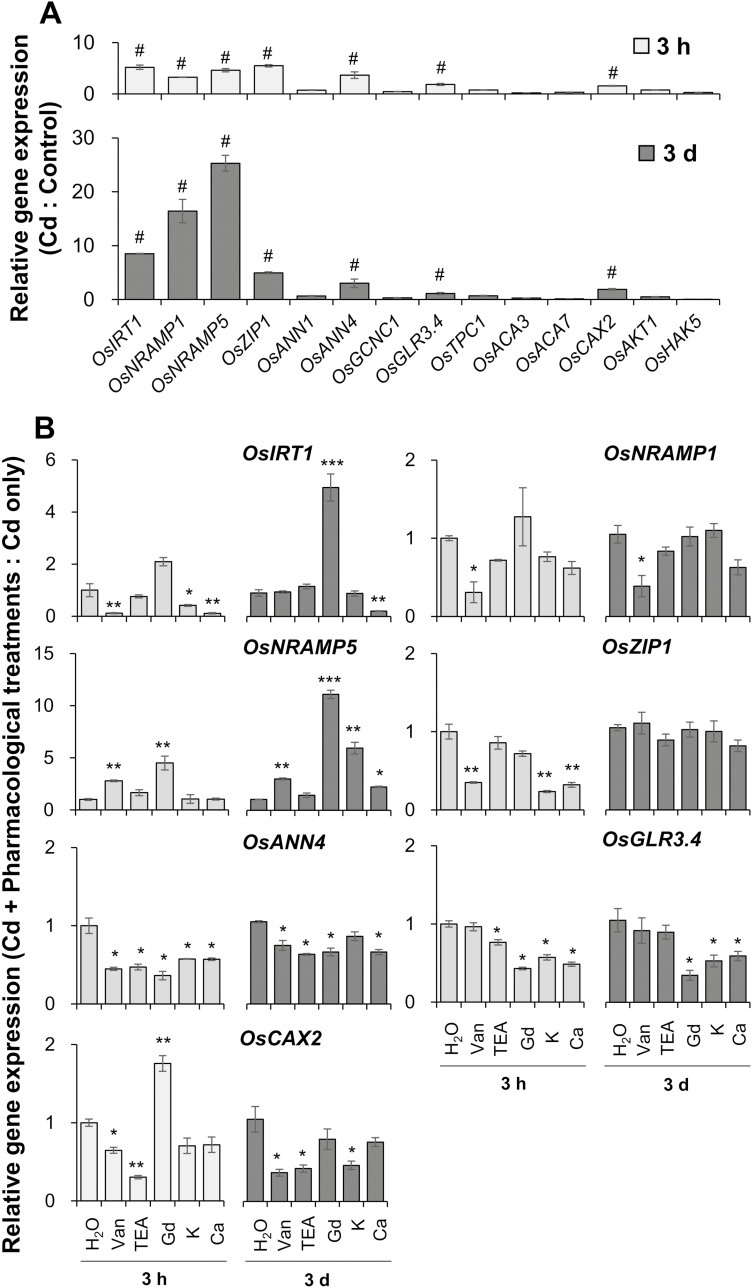
Gene expression of Cd transporters, Ca or K channels, and transporters under 20 µM CdCl_2_ without (A) or with (B) pharmacological treatments: 100 µM vanadate, 20 mM TEA^+^, 100 µM Gd^3+^, 10 mM K^+^, or 5 mM Ca^2+^ in the bulk roots. (A) Gene expression under Cd was compared relative to the control condition without Cd; (B) gene expression under Cd treatment plus pharmaceuticals was compared relative to the treatment with Cd only (H_2_O). Data are means ±SE (*n*=3 biological replicates). # in (A) represents the genes induced by 20 µM CdCl_2_; *, **, or *** in (B) represent the significance between the treatment with CdCl_2_ plus various pharmaceuticals and the treatment with CdCl_2_ only at *P*<0.05, 0.01, or 0.001.

As mentioned above, Cd^2+^ influx and uptake were significantly inhibited by the treatments with different pharmaceuticals (100 µM vanadate, 20 mM TEA^+^, and 100 µM Gd^3+^) and with elevation of Ca and K levels in the medium (5 mM CaCl_2_ and 10 mM KCl) (Figs 3–6). One possible reason for such an inhibitory impact of these pharmacological treatments on Cd^2+^ influx could be attributed to the significant reduction in Cd transport activity by the application of these pharmacological treatments. Therefore, the gene expression of four major plasma transporters for Cd, namely OsIRT1, OsNRAMP5, OsNRAMP1, and OsZIP1, was determined by qRT-PCR under treatment with 20 µM Cd with or without the pharmaceuticals. Under treatment with 20 µM Cd for 3 h and 3 d, the expression of *OsIRT1*, *OsNRAMP5*, *OsNRAMP1*, and *OsZIP1* was significantly induced (Fig. 8A). With the pharmaceuticals under the Cd condition, the expression of *OsIRT1* and *OsZIP1* was inhibited by the application of vanadate and the elevation of Ca and K in the medium, but such inhibition was nearly extinguished when the exposure time increased to 3 d (Fig. 8B). The expression of *OsNRAMP1* was significantly inhibited by the application of vanadate at both exposure times of Cd treatment (Fig. 8B). Surprisingly, however, the expression of *OsNRAMP5* was not inhibited but induced by the all of the above pharmacological treatments (Fig. 8B). These results indicated that the pharmacological treatment-induced (especially Gd^3+^-induced) inhibition in Cd^2+^ influx had little to do with the gene expression of the above four major plasma Cd transporters.

To examine the role of genes relevant to the pharmacological treatment-induced inhibition in Cd^2+^ influx, the transcript levels of 10 genes involved in transmembrane transport of Ca (*OsANN1*, *OsANN4*, *OsCNGC1*, *OsGLR3.4*, *OsTPC1*, *OsACA3*, *OsACA7*, and *OsCAX2*) and K (*OsAKT1* and *OsHAK5*) were also determined under treatment with 20 µM Cd with or without the pharmaceuticals. Results showed that three genes encoding Ca channels and transporters, namely *OsANN4*, *OsGLR3.4* and *OsCAX2*, were induced by the treatment with 20 µM Cd (Fig. 8A). The expression of *OsANN4* was inhibited by all the pharmacological treatments at 3 h and 3 d of Cd treatment (Fig. 8B). The expression of *OsGLR3.4* was significantly inhibited by the application of Gd^3+^ and the elevation of Ca and K in the medium at both exposure times of Cd treatment (Fig. 8B). On the other hand, the expression of *OsCAX2* was significantly inhibited by the application of vanadate and TEA^+^ at both exposure times of Cd treatment and the elevation of K at 3 d of Cd treatment (Fig. 8B). The expression of these three genes under the pharmacological treatments partially coincided with the results of Cd^2+^ influx and uptake (Figs 3–6), indicating a possible function for these proteins in transmembrane Cd transport.

## Discussion

### Cd uptake varies longitudinally along the rice root

In this study, the spatial kinetics of net Cd^2+^ flux across rice root cells was examined using the MIFE technique, which has a high spatial and temporal resolution and sensitivity to ion movement. In agreement with the previous microelectrode measurements in other plant species (Piñeros *et al.*, 1998; He *et al.*, 2011; Sun *et al.*, 2013), the present study found that the Cd^2+^ influx in roots of rice exposed to 20 µM CdCl_2_ was much greater in the root tip (0–2 mm from the root cap) than in the MZ (10–20 mm from root cap) (Figs 1, 2), which was further evidenced by the measurements of Cd concentration in root segments and the fluorescent labeling of Cd ions in the root tip, whether with or without pre-treatments (Figs 5, 6). Similar longitudinal variation in Cd^2+^ influx was also observed in sunflower roots using radioactive tracer techniques (Laporte *et al.*, 2013).

There may be several reasons to explain such longitudinal variation in Cd^2+^ influx. First, it may result from the alteration of root anatomy; that is, the development of Casparian bands and suberin lamellae in the endodermis and exodermis and cell lignification (Schreiber *et al.*, 1999; White, 2001; Laporte *et al.*, 2013). It has been documented that the presence of Cd produced Casparian bands, suberin lamellae, and lignification, which could restrict the apoplastic diffusion of Cd in root cells and consequently the whole-plant Cd accumulation (Enstone *et al.*, 2002; Lux *et al.*, 2004, 2011; Redjala *et al.*, 2011; Laporte *et al.*, 2013). In the present study, however, no significant difference in the development of Casparian bands and suberin lamellae and cell lignification was observed between the root tip (0–2 mm from root cap) and the MZ (10–15 mm from root cap) after 3 d of 20 µM CdCl_2_ (data not shown), indicating an inability of the apoplastic barrier to explain the occurrence of longitudinal variation in Cd^2+^ influx in this study. Another plausible reason for the longitudinal variation in Cd^2+^ influx may be attributed to the higher Cd transport activity of the root tip than the MZ, which could be reflected by the expression level of transporters for Cd. As expected, much higher expression of four well-known Cd transport genes, namely *OsIRT1*, *OsNRAMP1*, *OsNRAMP5*, and *OsZIP1* (Uraguchi and Fujiwara, 2012), was observed in the root tip than in the MZ, both prior to and after onset of Cd treatment (Fig. 7). These findings suggest that the cells of the meristem and EZs have stronger absorbing capacity for Cd uptake than the mature root cells, which might be attributed to the greater energy in that part of roots (Jones, 1998).

### Cd can enter into rice root cells via the pathways for Ca and K

A fundamental understanding of how Cd is taken up by rice roots would be a critical issue for revealing the mechanisms of Cd accumulation in rice grains. However, the non-essentiality and toxicity of Cd to plants make its entry into root cells a deep mystery. With years of effort, many transporters for essential elements, such as OsNRAMPs, OsZIPs, and OsIRTs, have been demonstrated to have an influx activity of Cd^2+^ in rice roots (reviewed by Uraguchi and Fujiwara, 2012, 2013). In the present study, our results revealed that the transcript levels of *OsIRT1*, *OsNRAMP1*, *OsNRAMP5*, and *OsZIP1* were indeed significantly induced by Cd treatment (Fig. 8A), proving their contribution to Cd uptake by rice roots. With the pharmacological treatments (100 µM vanadate, 20 mM TEA^+^, 100 µM Gd^3+^, 5 mM CaCl_2_, or 10 mM KCl) under Cd conditions, the expression of these four genes was either not changed or even up-regulated (Fig. 8B), especially for *OsNRAMP5* which is considered to be a major route of Cd uptake from the external environment and entry into cells in rice (Ishimaru *et al.*, 2012; Sasaki *et al.*, 2012). However, our MIFE and ICP-MS measurements revealed that the Cd^2+^ influx and uptake were significantly inhibited by the pharmacological treatments mentioned above (Figs 3–5), suggesting that these transporters only contribute in part to root Cd^2+^ influx and uptake, at least in the case where the external Cd concentration is as high as 20 µM in the present study (Lee and An, 2009; Takahashi *et al.*, 2014). Therefore, it can be assumed that there might be other pathways for Cd entry into rice root cells.

 It is well known that there is a competition between Cd^2+^ and Ca^2+^, because of their similarities in charge and ionic radius (Lindberg *et al.*, 2004; L.Z. Li *et al.*, 2012; S. Li *et al.*, 2012; Ahmad *et al.*, 2016). Many studies have reported that Cd may compete with Ca for uptake through ion channels in insects (Craig *et al.*, 1999), humans (Souza *et al.*, 1997), and plant guard cells (Perfus-Barbeoch *et al.*, 2002). The previous studies on Cd hyperaccumulators have also suggested that Cd could possibly enter the PM of root cells via Ca^2+^ channels (Lindberg *et al.*, 2004; Lu *et al.*, 2010; L.Z. Li *et al.*, 2012; Zhang *et al.*, 2017). To obtain an insight into the role of the Ca pathway in rice root Cd uptake, the present study investigated the competitive interactions of Cd and Ca with different techniques. The results of pharmacological measurements clearly showed that the elevation of the Ca level (from 0.1 mM to 5 mM) in the medium significantly inhibited root Cd uptake, in terms of both transient Cd^2+^ influx (Fig. 4; Table 1) and long-term total Cd uptake in rice roots (Figs 5, 6). These results suggested that Cd entry into rice roots was probably through Ca channels or transporters. Actually, several kinds of calcium-permeable channels, such as depolarization-activated calcium channels (DACCs), hyperpolarization-activated calcium channels (HACCs), and voltage-insensitive cation channels (VICCs), were reported to mediate Cd transport in plant roots (Lux *et al.*, 2011, and references therein). All of these channels are relatively non-selective between cations, and can be blocked by the NSCCs blocker Gd^3+^ (Lux *et al.*, 2011, and references therein). Indeed, the transient net Cd^2+^ influx of rice root in this study was completely blocked by Gd^3+^ (Fig. 3; Table 1) and the total Cd concentration in rice root was reduced by 58.1% (Fig. 5), implying that these non-selective channels may mediate Cd^2+^ transmembrane transport as it does for Ca^2+^ (S. Li *et al.*, 2012).

Potassium is the most abundant element in the plant cytosol and plays a vital role in nullifying the adverse impacts of stress on plants (Cakmak, 2005). Ahmad *et al.* (2016) reported that K supplementation could minimize the uptake of Cd. In the present study, the elevation of the K level (from 0.5 mM to 10 mM) in the medium was also observed to reduce the transient Cd^2+^ influx (Fig. 4; Table 1) and long-term total Cd uptake in rice roots (Figs 5, 6). These results indicated that the uptake of Cd into the cytosol of rice roots proceeds through channels permeable to K^+^. This was confirmed by the >95% reduction in the net Cd^2+^ influx (Fig. 3; Table 1) and 31.1% reduction in total Cd concentration (Fig. 5) of rice roots pre-treated with 20 mM TEA^+^, which is a well-known blocker of K^+^-selective channels (Maathuis and Sanders, 1996). A similar result was also found in the pharmacological measurements with fluorescent imaging in this study (Fig. 6) and in previous studies (Lindberg *et al.*, 2004). Unfortunately, no K channel or transporter has been found in plants yet to have the capacity to transport Cd^2+^, and the transcript levels of two well-known genes for transporting K to the cytosol (i.e. *OsAKT1* and *OsHAK5*) were not significantly induced by Cd treatment (Fig. 8A). However, one prokaryotic potassium channel isolated from *Methanobacterium thermoautotrophicum* named MthK, which contains a region called the regulator of the conductance of K^+^ (RCK) domain, has been found to bind divalent cations, such as Cd^2+^ and Ca^2+^ (Jiang *et al.*, 2002; Dvir *et al.*, 2010). It is also activated by Cd^2+^ more effectively than by Ca^2+^ (Kuo *et al.*, 2007). So, it could be hypothesized that some K channels, which have structural homology to the RCK domain of MthK, might have an ability to binding to Cd^2+^ similar to that of MthK. Indeed, several channels from higher plants, such as CASTOR and POLLUX in *Lotus japonicas* (Charpentier *et al.*, 2008), DMI1 (DOES NOT MAKE INFECTIONS1) in *Medicago truncatula* (Ané *et al.*, 2004), and SYM8 in *Pisum sativum*, have already been reported to share a short stretch of predicted structural homology with the pore region of MthK (Edwards *et al.*, 2007). All these channels are identified to bind Ca^2+^ and have the biological function of ion transmembrane transport. Yet there is no information reported yet on the possibility of these channels binding to and transporting Cd^2+^.

### Annexins and GLRs seems to be an important pool to explore the candidate channels participating in Cd absorption into rice root

It has been speculated that in Arabidopsis the gene families annotated as cyclic-nucleotide gated channels (CNGCs) and/or glutamate receptors (GLRs) are the most likely sources of genes encoding VICCs (Demidchik *et al.*, 2002; White, 2004; Swarbreck *et al.*, 2013). Moreover, it has also been suggested that the *AtTPC1* gene encodes a DACC (Furuichi *et al.*, 2001), and the annexin genes encode HACCs (White *et al.*, 2002). Thus, the expression level of several genes in these families and the other Ca transporter genes, namely *OsGLR3.4* (an ortholog of *AtGLR3.4*; Vincill *et al.*, 2012; Ni *et al.*, 2016), *OsCNGC1* (an ortholog of *AtCNGC1*; Nawaz *et al.*, 2014), *OsTPC1* (Kurusu *et al.*, 2004), *OsANN1* and *OsANN4* (LOC_Os01g31270 and LOC_Os05g31760; Jami *et al.*, 2012), *OsACA3* and *OsACA7*, and *OsCAX2*, which are involved in the transmembrance transport of Ca^2+^ (Singh *et al.*, 2014), were determined after onset of 20 µM Cd for 3 h and 3 d with or without pharmacological treatments. The results showed that three of them, *OsANN4*, *OsGLR3.4*, and *OsCAX2*, were induced by the treatment with 20 µM Cd (Fig. 8A). However, the expression of these genes was reduced by the application of pharmacological treatments to a different extent (Fig. 8B), partially coinciding with the results of Cd^2+^ influx and uptake (Figs 3–6), suggesting a possible function of these channels or transporters in transmembrane Cd transport. The response of annexin genes to Cd has been reported previously. Orthologs of *OsANN4*, such as *ANNAh3* in peanut (He *et al.*, 2015) and *ZmAnx9* in maize (Zhou *et al.*, 2013), were found to be induced by short (2–24 h) or long (14 d) Cd treatments. Furthermore, the ortholog of *OsCAX2* in Arabidopsis, *AtCAX2*, has been identified as a PM Cd transporter into root cells (Hirschi *et al.*, 2000). Therefore, the contribution of these channels or transporters to Cd absorption into rice root should not be ignored.

### Plasma membrane potential plays an important role in controlling transmembrane Cd transport

It is surprising that much greater inhibition of rice root Cd uptake was seen for Ca^2+^ and Gd^3+^ than for K^+^ and TEA^+^ (Figs 3–6; Table 1). Furthermore, a patch-clamp experiment on guard cells of fava bean demonstrated that Cd could permeate through Ca channels rather than K channels in guard cells (Perfus-Barbeoch *et al.*, 2002). These results suggest that Cd exhibits high affinity for Ca^2+^-binding sites over K^+^-binding sites during transport across the PM. In addition, pre-treatment with vanadate (a well-known inhibitor of PM H^+^-ATPase) also showed a significant impact on net influx (Fig. 3; Table 1) and the total uptake (Figs 5, 6) of Cd^2+^ in rice roots, although its inhibitory effect was much less compared with the other pharmacological agents used in the present study, indicating that the uptake of Cd into root cells is partly dependent on membrane potential. So, it cannot be excluded that the pharmaceuticals in this study may inhibit the uptake of cadmium by depolarizing the PM, as they all can trigger membrane potential depolarization (Huddart and Hill, 1996). It has been reported that high K^+^ in the medium was likely to depolarize the membrane by opening high conductance K^+^ channels (Beilby, 1985), and high Ca^2+^ in the medium could reduce background conductance which was thought to be mediated by the NSCCs (Shepherd *et al.*, 2008). Therefore, it would be expected that Cd may enter root cells via hyperpolarized ion channels, for example the HACCs, such as *OsANN4* in the present study, like those described in previous studies (Perfus-Barbeoch *et al.*, 2002; Lux *et al.*, 2011; L.Z. Li *et al.*, 2012).

In conclusion, Cd uptake exhibited a clear longitudinal variation along rice roots, with the root tip (including the meristem and EZs) showing much higher Cd^2+^ influx and Cd concentration than the MZ, which might be attributed to the much higher gene expression of *OsIRT1*, *OsNRAMP1*, *OsNRAMP5*, and *OsZIP1* in comparison with the MZ. The Cd^2+^ uptake by rice root cells was restricted by the blockage of ion channels and the elevated external levels of K^+^ and Ca^2+^, regardless of Cd exposure time, suggesting an important role for ion channels permeable to cations such as K^+^ and Ca^2+^ in transmembrane Cd transport. The Cd transporters OsIRT1, OsNRAMP1, OsNRAMP5, and OsZIP1 are in part responsible for the Cd^2+^ entry into rice root, and the Ca channels OsAAN4 and OsGLR3.4 might play an important role in rice root Cd uptake. However, the permeability and selectivity of these channels to Cd^2+^ and their value in ‘low-Cd’ rice innovation need to be further elucidated using electrophysiological and molecular techniques.

## Supplementary data

Supplementary data are available at *JXB* online.

Table S1. Primers for qRT-PCR.

Supplementary Table S1

## Abbreviations

**Abbreviations**
Ca: calcium
Cd: cadmium
CNGC: cyclic-nucleotide gated channel
DACC: depolarization-activated calcium channel
EZ: elongation zone
GLR: glutamate receptor
HACC: hyperpolarization-activated calcium channel;
K: potassium
LCT: low affinity calcium transporter
MIFE: microelectrode ion flux estimation
MZ: mature zone
NRAMP: natural resistance-associated macrophage protein
NSCC: non-selective cation channel
PM: plasma membrane
TEA^+^: tetraethylammonium
VICC: voltage-insensitive cation channel
YSL: Yellow-Stripe 1-Like
ZIP: zinc/iron-regulated transporter-like protein
