# Supplementary material for: The pathway of transmembrane cadmium influx via calcium-permeable channels and its spatial characteristics along rice root
Source: J Exp Bot. 2018 Aug 7;69(21):5279–91. doi: 10.1093/jxb/ery293 (PMC6184580; doi:10.1093/jxb/ery293)
Supplement: Supplementary Table S1 [file ery293_suppl_supplementary_table_s1.pdf]

Supplementary Table S1 Primers for qRT-PCR

Function	RGAP locus ID	Gene name	Primer sequence (5'-3', forward / reverse)
Cd transporters	LOC_Os03g46470	*OsIRT1*	CGTCTTCTTCTTCTCCACCACGAC / GCAGCTGATGATCGAGTCTGACC
	LOC_Os07g15370	*OsNramp5*	AAGAGGACGCCGACAAGTG / TGTGCCGGTAATAGTGGAGC
	LOC_Os07g15460	*OsNramp1*	GGATTCTCCTGGGTGCTGGGGTT / GCAACAATCTACTCCCATGGGCC
	LOC_Os07g12890	*OsZIP1*	ATTCTGTTGCAAGTTCGGCG / AGTCAATTGCCTGTAGCTCTCC
Ca Channels	LOC_Os01g31270	*OSANN1*	GGCCGGAAGTCAACACAAAG / GCCTTGAGATCCTTGTTGATTGG
	LOC_Os05g31760	*OsANN4*	TCTTTGCCAAACCCTGTTGC / TCGTCTCTTCCACATGAACTGG
	LOC_Os01g57370	*OsCNGC1*	AGTAATGCAGCTAGAAATAAC / CAAAACTAGACCTGATGTCGA
	LOC_Os06g13730	*OsGLR3.4*	ACCTGATGAGCCCAAAGACG / ACTGCGCATCTGAAGAACCA
	LOC_Os01g48680	*OsTPC1*	TGGCAAGCTTGGATGGAGAG / CCATGGTCCCCTTTGTCCTC
Ca transporters	LOC_Os03g10640	*OsACA3*	TGACAAGCACACCTTGGTGAA / CATCACCAGTAACCGCAACAA
	LOC_Os10g28240	*OsACA7*	GGTGGGCATGATTGATCCGAG / ACGGTGCGAACCCTCTTG
	LOC_Os02g04630	*OsCAX2*	GATGTTGTTGTACTGATGCC / CTGGCACCGTTTGGAAATGT
K Channel	LOC_Os01g45990	*OsAKT1*	TCTACTCTGCGTGGGTCTCA / CGCCATGCGATCTTCTTTGG
K transporter	LOC_Os01g70490	*OsHAK5*	GCATACGGTATTGCTGTTGTC / TGGCAAGTATCCACCTTGAGT
		OsActin	GCATCTCTCAGCACATTCCA / GCGATAACAGCTCCTCTTGG
